# The effects of Phoniatric PREhabilitation in Head and Neck Cancer patients on Aspiration and Preservation of Swallowing (PREHAPS): study protocol of a monocentric prospective randomized interventional outcome-blinded trial

**DOI:** 10.1186/s13063-024-08010-2

**Published:** 2024-03-22

**Authors:** Julian Kuenzel, Stephan Duerr, Sarah Vester, Florian Zeman, Gunnar Huppertz, Michael Koller, Gerda Pfleger, Annika Woertgen, Hazem Salloum, Monika Klinkhammer-Schalke, Tobias Pukrop, Peter Kummer

**Affiliations:** 1 Department of Otorhinolaryngology, University Hospital Regensburg, Franz-Josef-Strauß-Allee 11, Regensburg, 93053 Germany; 2 Department of Otorhinolaryngology, Section Phoniatrics and Pediatric Audiology, University Hospital Regensburg, Regensburg, Germany; 3 Center for Clinical Studies, University Hospital Regensburg, Regensburg, Germany; 4 Institute for Quality Management and Health Services Research, University Hospital Regensburg, Regensburg, Germany; 5 Department of Internal Medicine III, Hematology and Oncology, University Hospital Regensburg, Regensburg, Germany

**Keywords:** Prehabilitation, Head and neck cancer, Swallowing, Dysphagia, Aspiration, FEES, Phoniatrics, Quality of life, Randomized controlled trial

## Abstract

**Background:**

Dysphagia, with its negative impact on life expectancy and quality of life, is a major side effect of head and neck squamous cell carcinoma (HNSCC). In a typical Head and Neck Cancer Center, more than half of patients are affected. Improving treatment, and ideally prevention respectively prehabilitation, therefore seems more than desirable.

**Methods:**

The study is planned as a monocentric, prospective, outcome-blinded, randomized interventional study comparing an advanced phoniatric-logopedic prehabilitation with a control (standard of care). Seventy patients (30 control group, 30 intervention group, 10 drop-out rate of 15%) with an initial diagnosis of invasive HNSCC and curative treatment intention will be included over a period of 17 months. In addition to the previous standard, both groups will undergo both detailed subjective assessment of swallowing function and quality of life by means of various questionnaires and objective analyses by bioelectrical impedance measurements and phoniatric endoscopic swallowing examinations. In the intervention group, risk-related nutritional counseling (face-to-face) and phoniatric-logopedic prehabilitation are provided: detailed counseling with video demonstration and exercises to strengthen and improve the range of motion of the oral, pharyngeal, and laryngeal muscles (guided by exercise diary). Controls are performed at 6 weeks, 3 and 6 months, and 9 or 12 months after the end of therapy during the regular tumor follow-up. Primary study endpoints are swallowing function and emotional distress at 6 weeks of control visit.

**Discussion:**

Prehabilitation measures have already proven successful in other patient groups, e.g., transplant patients. In the field of head and neck oncology, interest in such concepts has increased significantly in recent years. However, usually, only subgroups, e.g., patients with swallowing problems after radiochemotherapy alone, are in focus. Our study aims to investigate the general benefit of prehabilitation with regard to swallowing function, which is so important for protection of aspiration and quality of life.

**Trial registration:**

German Clinical Trials Register DRKS00029676. International Clinical Trials Registry Platform DRKS00029676. Registered on 19 July 2022.

**Supplementary Information:**

The online version contains supplementary material available at 10.1186/s13063-024-08010-2.

## Background

Head and neck squamous cell carcinoma (HNSCC) is the sixth most common malignant tumor disease, mostly affecting the upper aerodigestive tract [1]. At a typical Head and Neck Cancer Center, approximately 55% of patients currently suffer from dysphagia [2]. In subgroups with, e.g., oropharyngeal carcinomas, such disorders occur in up to 80% of cases, fatal because in this group the proportion of younger patients is increasing worldwide due to the association with HPV (human papillomavirus) and already reaches 30–40% in Germany [3]. Especially these younger patients rightly expect special attention to swallowing disorders because of disease and therapy. Last, but not least, clinically relevant anxiety or depression symptoms are regularly associated with dysphagia, in almost 50% of cases [4], hardly surprising considering the central social importance of eating and drinking together. Elderly patients (over 65 years of age) are at mortality risk from dysphagia, especially from aspiration pneumonia [5]. Often, patients with HNSCC have low socioeconomic status and relevant comorbidities that lead to retirement in up to 50% of cases after therapy [6]. In contrast, early rehabilitation of dysphagia appears to mitigate the financial consequences of the disease [7]. Swallowing disorders in head and neck tumor patients are thus highly relevant for effective therapeutic interventions not only because of the directly associated limitations of quality-of-life (QoL), but also because of the risk of complications.

In Germany, the focus is on post-therapeutic rehabilitation, and the treatment of preexisting swallowing disorders disregards preventive efficacy, both before and after oncological therapy. The introduction and implementation of a complex pre-therapeutic phoniatric intervention (diagnostics by means of endoscopic examination of swallowing function as well as comprehensive questionnaires and patient self-reports; therapy by means of individual logopedic counseling with instructions for exercise treatment of swallowing function) as well as a comprehensive malnutrition screening with nutritional counseling will therefore be tested in a study in the setting of a University Head and Neck Cancer Center in Germany. The benefit of such a measure prior to treatment of a representative cohort of HNSCC patients is currently unknown, as is any harm. In the context of post-therapeutic rehabilitation, logopedic swallowing therapy is of great importance, whereas a harmful effect has not been demonstrated here either.

We expect that this approach will improve swallowing function and also reduce the emotional burden of patients, so that not only the oncological outcome, but also the peri- and post-therapeutic QoL can be significantly improved.

## Methods

### Study design

The study is designed as a monocentric, prospective, randomized (parallel group; allocation ratio 1:1), outcome-blinded, controlled interventional trial (exploratory RCT) at the Head and Neck Cancer Center of the University Hospital Regensburg. The intervention consists of individual information and risk consulting, individual logopedic counseling with instructions for exercise treatment of swallowing function, and risk-related nutritional counseling (face-to-face). In the control group, patients are treated according to the currently applicable standard of care described in the guidelines on laryngeal cancer [8] and oral cavity cancer [9] as well as in the survey on Head and Neck Cancer Centers of the German Cancer Society (https://www.krebsgesellschaft.de/zertdokumente.html).

### Outcomes

All patients participating in the study are analyzed by means of various questionnaires in addition to the therapy standard of the Head and Neck Cancer Center Regensburg:

- EORTC-QLQ-C30 for the general assessment of QoL [10]
- EORTC-QLQ-HN43 to assess the QoL of patients with HNSCC [11]
- Dysphagia Handicap Index (DHI) for measuring the handicapping effect of dysphagia on the physical, functional, and emotional aspects of people’s lives [12]
- Hospital Anxiety and Depression Scale (HADS) for assessing anxiety and depression [13]
- Functional Oral Intake Scale (FOIS-G) to analyze oral food intake [14]

In addition, a bioelectrical impedance analysis, a determination of the body mass index (BMI), and measurement of serum albumin level take place.

Furthermore, a detailed phoniatric diagnosis of swallowing (Functional Endoscopic Evaluation of Swallowing (FEES)) regarding restrictions of oral nutrition and aspiration risk, which is objectively evaluated by means of the Penetration-Aspiration Scale (PAS) [15] and the Yale Residue Scale (YRS) [16], is performed.

Primary study endpoints, 6 weeks after the end of therapy:

- Swallowing function (objectively assessed using FEES: PAS, YRS, subjectively using DHI and FOIS-G)
- Emotional distress: anxiety and depression (HADS)

Secondary study endpoints 6 weeks, 3 and 6 months, and 9 or 12 months after the end of oncologic therapy:

- Swallow-related QoL (DHI; EORTC-QLQ-HN43; FOIS-G).
  - ◦ Time to decannulation
  - ◦ Percentage of nutrition by gastric feeding tube/time of dependence on enteral nutritional substitution via a gastric feeding tube
  - ◦ Incidence of complications (aspiration pneumonia)
- Inpatient length of stay, incl. readmission
- Nutritional status (BMI, albumin, bioelectrical impedance analysis)
- General QoL (EORTC-QLQ-C30)
- Occupational reintegration
- Subgroup analyses regarding older patients (> 65 years) and patients with weaker social status

#### Eligibility criteria of study participants

Inclusion criteria:

- Initial diagnosis of invasive HNSCC

Exclusion criteria:

- T1 glottic carcinoma, salivary gland tumors, sinus, nasal cavity carcinomas, lip carcinomas, skin carcinomas
- Planned laryngectomy, total glossectomy, esophageal dysphagia
- No curative therapy
- State after therapy of carcinoma of the upper aerodigestive tract or esophageal carcinoma
- State after radiotherapy in the head and neck region
- Higher-grade cognitive impairment (e.g., dementia, Korsakow syndrome)
- Psychomotor impairment (e.g., Parkinson's disease), previous neurological diseases with dysphagia (e.g., post apoplexy)
- Age < 18 years
- ECOG > 2
- Patient is unable to complete questionnaires even with assistance (inadequate ability to read and write, higher grade inadequate hearing loss).
- Language barrier
- Pregnancy/breastfeeding

### Interventions

For a detailed analysis of the effects of Phoniatric PREhabilitation in Head and Neck Cancer patients on Aspiration and Preservation of Swallowing, two study arms were designed (Table 1). In the intervention group, additional measures take place compared to the control group:

- General information and risk counseling on the swallowing examination, also for the prevention of dysphagia before and during therapy with video demonstration [17], if necessary, also to accompanying relatives.
- Logopedic exercises to strengthen and improve the sensitivity and range of motion of oral, pharyngeal, and laryngeal muscles (tongue, larynx, jaw, e.g., Mendelsohn maneuver, Shaker maneuver, effortful swallow, supraglottic swallowing technique). The exact therapeutic procedure is based on an individual protocol. The patient is instructed to discontinue or modify in-home exercises (eating rules, individual exercise protocol with recommended dosage, documentation in exercise diary). The prescribed dose of exercise treatment takes into account not only the need but also the expected patient’s adherence to therapy. It includes at least 10 repetitions 3 times a day for each of, e.g., 5 forms of exercises [4], so that a total dose of 150 exercises per day is achieved. Even in the case of minor symptoms, preventive counseling and exercise treatment are carried out with a view to the future. After 3–7 days, a speech therapist conducts a control interview (face-to-face or by telephone) based on the exercise diary. If it seems necessary, an intervention can take place at short notice.
- Additional nutritional risk screening [18] and risk-related nutritional counseling (face-to-face), performed by staff of the Department of Internal Medicine III, Hematology and Oncology.

**Table 1:** Study design—intervention versus control group

Measures’ description	Study arms	
	**Intervention**	**Control**
**Pre-therapeutic standard**		
Standardized non-phoniatric, medical assessment of swallowing function, if necessary phoniatric-logopedic diagnostics and therapy according to standard; in some cases low-frequency therapy already before/during therapy, usually by cost adaptation	X	X
Tumor board	X	X
Medical information (diagnosis, surgery or radiotherapy)	X	X
Psycho-oncological screening (Hornheider questionnaire), intervention if necessary	X	X
Contact and advise social service	X	X
PEG, if necessary	X	X
Nutritional Risk Screening (NRS)—prescreening in nursing history	X	X
Proactive information about smoking cessation (flyer)	X	X
**Baseline values (both groups, in addition to the previous standard)**		
EORTC-QLQ-C30 [10], EORTC-QLQ-HN43 [11], Dysphagia-Handicap-Index (DHI) [12], Hospital Anxiety and Depression Scale (HADS) [13]	X	X
NRS main screening, risk-related nutritional counseling (face-to-face); bioelectrical impedance analysis	X	X
Phoniatric diagnostics (FEES) regarding oral feeding restrictions and aspiration risk: Penetration Aspiration Scale (PAS) [15]; Functional Oral Intake Scale (FOIS-G) [14]; Yale Residual Scale [16]	X	X
**Intervention**		
Phoniatric-logopedic PREhabilitation General information and counseling, also for prevention of dysphagia before/during therapy, video demonstration [17], if necessary also by accompanying relatives, exercises to strengthen and improve the range of motion of oral, pharyngeal, and laryngeal muscles (tongue, larynx, jaw, e.g., Mendelsohn, Shaker maneuvers, “effortful swallow,” supraglottic swallowing technique) and sensitivity. Therapeutic approach according to individual protocol. Instructions for home exercise (eating rules, individual exercise protocol with recommended dosage, instructions for exercise diary)	X	-
Follow-up interview, based on exercise diary, after max. 1 week, by phoniatrics, speech therapy and nutrition counseling, face-to-face or by telephone	X	-
**Oncological therapy**		
**Postinterventional outcome measures at 6 weeks and 3, 6, and 12 months after the end of oncologic therapy**		
Questionnaires: EORTC-QLQ-C30 [10]), EORTC-QLQ-HN43 [11]), FACE-Q Head and Neck Cancer [19]), Dysphagia-Handicap-Index (DHI) [12]), Hospital Anxiety and Depression Scale (HADS) [13])	X	X
Phoniatric-logopedic follow-up (FEES) regarding oral feeding restrictions and aspiration risk, Penetration Aspiration Scale (PAS) [12]; Functional Oral Intake Scale (FOIS-G) [14]; Yale Residual Scale [13] and on the progress of therapy (exercise diary)	X	X
**Follow-up treatment (AHB) after 6 weeks, according to patient preference**		

### Sample size and recruitment

Since this type of therapeutic approach was not studied yet in this form, it was not possible to estimate an expected effect size for a sample size calculation. Therefore, the study design for the present project was chosen with a complex family of endpoints to obtain first estimates of treatment effects for the clinically relevant endpoints with the consequence that no sample size calculation based on an expected treatment effect of one primary endpoint was performed. Instead, sample size considerations are based on assumptions about possible effect sizes of different endpoints (primary endpoint family) with the aim to get reliable and valid effect estimates for a following more extensive multicenter clinical trial. After sharpening the focus, the results of this study will be used to initiate a multicenter confirmation study, e.g., within the framework of the BZKF (Bavarian Center for Cancer Research, Head and Neck Tumor Study Group). According to the publication by Guillen-Sola [20] we assume median effect sizes within the primary endpoint family. They state an effect in the Penetration-Aspiration-Scale of 2 Units and a difference ≥ 10 units in the tests of quality of life (EORTC-QLQ-C30 and EORTC-QLQ-HN35). This is also the expectation of the therapy based on our clinical experience. Here, a “median effect size” methodologically means a Cohen’s *d* of approximately 0.5 or half a standard deviation [21]. 

To obtain sufficient accuracy for the estimation of treatment effects due to the performed intervention as well as for the planning of a confirmative follow-study, *n* = 30 patients per study arm (*n* = 60 in total) are to be analyzed under the assumption of median effect sizes regarding the endpoints of the primary endpoint family [22]. Assuming a drop-out rate of 15%, *n* = 70 patients have to be included and randomized in the study. In the recruitment period of 17 months, we assume a potential study population of 350 patients (250 primary cases in the Head and Neck Cancer Center per year). Considering the increase in higher tumor stages due to the corona pandemic, we expect 40% of patients (*n* = 140) to meet all inclusion and exclusion criteria, of which 50% of patients (*n* = 70) are likely to give informed consent. Thus, inclusion of 70 patients in the planned recruitment period is realistic. Screening and identification of potential study patients will occur during the patient’s first inpatient stay (primary diagnosis, histology acquisition, pan-endoscopy, staging). After the tumor diagnosis has been confirmed, the first contact with the potential study patient is made in the Ear, Nose and Throat Clinic and the Clinic for Oral and Maxillofacial Surgery (usually after the interdisciplinary tumor board) by physicians from the clinics with the support of the study assistance. The detailed study information as well as the study inclusion is carried out by the medical staff of the Section of Phoniatrics and Pediatric Audiology. 

The study is explained in detail to the patient by a physician from the Section of Phoniatrics and Pediatric Audiology. Subsequently, the patient gives an informed consent to participate in the study by means of a written declaration of consent. As part of the consent process, the participant will be asked whether the data collected may also be used for questions unrelated to the study but related to the study purpose. The participant can agree to such use or refrain from doing so. Randomization is performed prior to the intervention, after informed and signed consent, via REDCap database. Each randomization is documented and signed by the investigator.

### Statistical analysis

The primary outcome measure is a complex family of endpoints including swallowing function (subjectively using DHI, FOIS-G, and objectively using FEES) and anxiety and depression (HADS) 6 weeks after the end of therapy. Each questionnaire score will be calculated using the associated manual. The two study groups will be compared for each endpoint using analysis of covariance (ANCOVA). The respective endpoint at the time point after 6 weeks is included in the model as the dependent variable, the study group as factor, and the respective baseline value at baseline as covariate. To check the endpoints, the intention-to-treat collective will be used as the evaluation collective. Analyses will also be evaluated on the per-protocol collective as a control. Established, clinically relevant effect sizes will be used as a comparison to evaluate the effects (estimated marginal means and 95% confidence intervals) and their benefit to patients. A panel of experts (phoniatrics, ENT, maxillofacial surgery, speech therapy, study assistance, biometry) will evaluate which changes in primary outcome parameters should be interpreted as clinically relevant. In addition, subgroup analyses will be performed regarding older patients (> 65 years) and patients with weaker social status. The concept of complex pre-therapeutic phoniatric prehabilitation is considered promising if there is a consistent advantage over the control group within the complex family of outcomes.

Secondary study endpoints 6 weeks, 3 and 6 months, and 9 and/or 12 months after the end of oncologic therapy will be considered exploratively: swallowing-related QoL, subjectively perceived swallowing function, incidence of aspiration pneumonia, general QoL, nutritional status, time to decannulation, nutritional percentage by gastric feeding tube, time of dependence on enteral nutritional substitution via gastric feeding tube, duration of inpatient stays, and occupational reintegration.

The analysis of secondary endpoints is purely descriptive and exploratory. Depending on the type of endpoint, covariance analyses for metric endpoints, logistic regressions for dichotomous endpoints, or simple tests such as Student’s *t*-tests, Wilcoxon Mann–Whitney *U* tests, or chi-square tests are used.

### Randomization and blinding

#### Randomization

Randomization of a patient is stratified according to UICC stage (8th edition) into stratum I (UICC I and II) and stratum II (UICC III and IV). This considers that patients treated by surgery alone are generally assigned to the earlier tumor stages UICC I and II and are less likely to require adjuvant therapy. Patients with an indication for adjuvant radiotherapy or radiochemotherapy or primary radiotherapy or simultaneous radiochemotherapy are more likely to belong to the advanced tumor stages UICC III and IV. Randomization will be computerized (REDCap database) by a physician from the Section of Phoniatrics and Pediatric Audiology prior to intervention, after informed and signed consent (see above).

#### Blinding

The study is designed outcome-blinded, meaning that each detailed phoniatric swallowing examination (FEES) is subsequently assessed again objectively by another physician from the Section of Phoniatrics and Pediatric Audiology based on the video documentation using the specified documentation parameters (PAS, YRS). The second assessor does not know whether the examined patient is from the control or intervention group. Unblinding is not provided.

### Trial flow

**Fig. 1:**
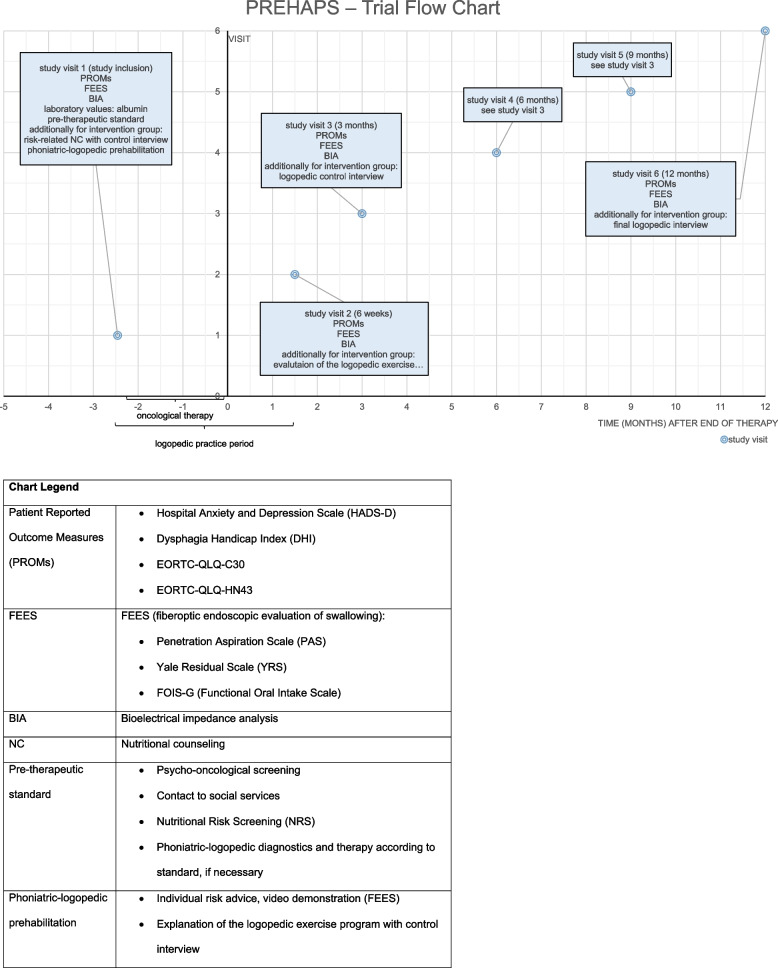
PREHAPS—trial flow chart

In the intervention group, the inclusion examination is followed by nutritional counseling and a first control interview by a speech therapist based on the exercise diary taking place in the presence or on the phone after approximately 3–7 days. Further appointments are scheduled at 6 weeks, 3 and 6 months, and 9 or 12 months after the end of oncological therapy (Fig. 1).

There will be no special post-trial care, but the standard surveillance program.

### Data management

In this study, the Research Electronic Data Capture (REDCap) system is used to implement electronic case report forms (eCRFs). All participant data throughout the study will be collected and directly entered into eCRFs. Data entries will be time-stamped and user-tracked, providing a detailed audit trail for monitoring data changes.

The randomization module of REDCap will be used to assign participants to one of both study groups (intervention/control group). The system’s ability to implement complex randomization algorithms facilitated the stratification process, ensuring a balanced allocation of participants across different study groups and strata.

### Quality assurance

#### Monitoring

Onsite monitoring follows a risk-based approach and is laid down in a monitoring plan. 100% SDV include informed consent and randomization. Within the established data management process, specific data quality rules are applied to collected data. Any deviation, unexpected missing, or implausibility of data will be reported to the study site for clarification.

#### Data monitoring committee

The data monitoring committee (DMC) is a panel of experts (phoniatrics, ENT, maxillofacial surgery, speech therapy, study assistance, biometry) and will evaluate which changes in primary outcome parameters should be interpreted as clinically relevant (see above). Interim analyses are not provided.

## Discussion

### Innovation content of the study

To the best of our knowledge, the described study is the first in Germany to address a pre-therapeutic swallowing intervention for patients with head and neck cancer. The feasibility (practicability, patient acceptance, etc.) and the integration of this complex and interdisciplinary intervention into the pre-therapeutic care process are investigated. The patient population is typical for a German Head and Neck Cancer Center including a high proportion of patients treated with primary surgery in addition to patients receiving primary radiation. Guideline recommendations concerning early phoniatric intervention in HNSCC patients are integrated insufficiently into the care process so far [8, 9, 23].

### Relevance of the study

The main pillars of primary therapy for HNSCC are, on the one hand, primary surgical procedures with functional reconstruction if necessary, often followed by adjuvant radiotherapy, and, on the other hand, primary radio-(chemo)therapy. Due to the critical localization of tumors in the upper aerodigestive tract, they lead to hoarseness as a relatively early symptom and/or often impair swallowing function. Swallowing dysfunction not only significantly reduces the QoL of patients, but also leads to life-threatening complications such as aspiration pneumonia in up to 25% of cases [24]. An analysis of the SEER cancer registry combined with US health insurance data showed a 3- to fourfold higher incidence of aspiration pneumonia for patients with HNSCC treated by primary radiochemotherapy compared with a control group of the same comorbidity. The hospitalization rate was 84%, and intensive therapy was required in nearly half of the cases. The relative risk of death increased up to 42% and there was a 15% reduction in 5-year survival [10].

A recent study of our group showed that existing postoperative dysphagia with an inability to take in oral food is a very early indicator of poor survival, irrespective of the tumor stage [23]. In addition, prolonged dysphagia regularly leads to malnutrition, sometimes with significant weight loss [25]. The prognostic significance of malnutrition lies in the prediction of early mortality and early recurrence, as well as a cellular immune deficiency, which limits curative therapy options even in times of immuno-oncological therapies [26]. For the differentiated examination of patients who are malnourished despite an inconspicuous BMI, measurement tools such as nutritional risk screening have already been introduced into standard oncological care or can be supplemented by modern methods such as bioelectrical impedance analysis [27].

A Canadian metanalysis provides initial evidence for the benefit of preventive treatment of dysphagia in primary irradiated head and neck tumor patients, although the results are inconclusive regarding approach, type of therapy, proper timing, and dose [12, 20, 27–29]. Surgical therapeutic procedures, as is the widespread standard of care in Germany, are poorly represented in the literature [27, 30]. Similarly, a Cochrane systematic review and another review revealed great heterogeneity in terms of study design, methodology, and the effectiveness of prophylactic swallowing exercises and encouraged further methodologically well-designed studies to better classify methods and their outcome measurement [4, 31]. Recent studies from other healthcare systems, such as the English NHS, have already demonstrated the feasibility of pre-therapeutic swallowing intervention in patients with advanced tumor stages (UICC III/IV) [32].

In many cases, however, patients are discharged from oncological care who are exclusively or substantially dependent on non-oral forms of nutrition, burdened with an uncertain prospect of at least partial recovery of natural function. The focus on post-therapeutic rehabilitation and separation from the care at the oncological centers combines restrictions on the individual possible therapy success with the danger of a reduced use of rehabilitative measures, because of a lack of education and motivation of the patients.

According to clinical experience, such preventive rehabilitative approaches are almost completely lacking in routine care in Germany [33]. Although the therapy of head and neck tumors at oncological centers today usually achieves what is necessary and possible, the rehabilitation of functional deficits is shown to be comparatively incomplete. The benefit of pre-therapeutic evaluation and intervention is hinted at in the current AWMF (*Arbeitsgemeinschaft der Wissenschaftlichen Medizinischen Fachgesellschaften e. V.*) organ guidelines, the S3 guidelines on oral cavity carcinoma [9] and laryngeal carcinoma [8], but recommendations regarding approach, type of therapy, timing, and dose remain vague. The preoperative or pre-therapeutic preparation of patients for these expected therapeutic consequences thus remains inadequate.

Thus, there is an urgent need to evaluate the existing evidence for the potential benefit of pre-therapeutic phoniatric intervention in a study setting with a realistic outcome model at a German Head and Neck Cancer Center.

### Improvement of care

The introduction of rehabilitative elements of pre-therapeutic phoniatric intervention, at the earliest possible time before and during acute therapy, has considerable potential to improve care:

- Improvement of swallowing function represents, in the first place, a significant contribution to quick recovery and a crucial contribution not only to QoL (regaining oral nutrition and social reintegration, especially of socially disadvantaged patients), but also to patient safety (prevention of malnutrition and aspiration pneumonia).
- Suffering and burden of patients with head and neck tumors and the high prevalence of swallowing disorders demonstrate the great need. Data in the literature indicate a high appropriateness of the planned intervention and suggest a relevant improvement in routine care.
- Information about dysphagia as a consequence of disease and therapy first contributes significantly to patient education, not only to identify existing disorders, but also to name expected disorders and to offer therapeutic options, to assess their nature, possibilities, and limitations in a very specific way in each individual case. This education thus makes a significant contribution to participation; in combination with an exercise program, it strengthens self-efficacy (empowerment).
- With greater efficiency and time economy, the application and adaptation of existing OPS codes (8–553) for prehabilitation, which are graded according to the amount of work involved, would also make sense from a cost-economic point of view.
- The cost-effectiveness of the statutory health insurance (SHI) system enhances in two ways, on the one hand by improving the oncological outcome (reduction of morbidity of aspiration pneumonia) and the functional outcome (regaining oral nutrition and social reintegration), and on the other hand by cost savings (costs for non-oral nutrition, inpatient treatment costs, including intensive care).

### Utilization potential

From the perspective of the SHI system, cost-saving potentials are of considerable interest. If the concept of prehabilitation proposed here proves successful, these will result in a reduction in non-oralization (costs of gastric feeding), complications (aspiration pneumonia), and inpatient stays. In sum, this may lead to an increase in the rate of occupational reintegration, which is of considerable importance, especially in patients of socially weaker status. For both increasingly younger and older patients, a successful concept of prehabilitation suggests an improvement in overall outcome, which should be reflected in the alleviation of individual suffering, reduction in family stress, and relief for society as a whole. After sharpening the focus, the results of the proof-of-concept study should be used to initiate a multicenter confirmation study, e.g., within the framework of the BZKF (Bavarian Center for Cancer Research, Head and Neck Tumor Study Group) or even nationwide programs. In addition, it is conceivable that phoniatric prehabilitation can also be offered across sectors in an outpatient setting, for example involving logopedic and ENT specialist practices, prior to the start of oncological therapy. The results of the study will also be used to further develop guidelines in this field within the national AWMF system.

## Conclusion

The aim of our study is to assess the general benefit of prehabilitation of swallowing disorders in patients with head and neck tumors at a University Head and Neck Cancer Center. Dysphagia affects patients’ quality of life, leads to life-threatening complications such as aspiration pneumonia, and may be the cause of malnutrition or cachexia. Dysphagia is also a significant problem from a public health perspective. Thus, it is reasonable and necessary to scientifically investigate possible measures for the prevention and treatment of swallowing disorders in patients with head and neck tumors.

## Trial status

Protocol version number: 2, 16.12.2021

Start of recruitment: 01.07.2022

End of recruitment (approx.): 31.03.2024

### Supplementary Information

**Supplementary Material 1.**

## Data Availability

Not applicable.

## Abbreviations

AWMF: *Arbeitsgemeinschaft der Wissenschaftlichen Medizinischen Fachgesellschaften e. V.*
BMI: Body mass index
DHI: Dysphagia Handicap Index
eCRF: Electronic case report forms
FEES: Functional Endoscopic Evaluation of Swallowing
FOIS-G: Functional Oral Intake Scale
HADS: Hospital Anxiety and Depression Scale
HNSCC: Head and neck squamous cell carcinoma
HPV: Human papillomavirus
PAS: Penetration-Aspiration Scale
QoL: Quality of life
SHI: Statutory health insurance
YRS: Yale Residue Scale
